# The distinct characteristics of the endophytic microbiome and metabolome within the root system result in varying abilities of watermelon resistance against *Fusarium* wilt

**DOI:** 10.1186/s12870-025-07488-5

**Published:** 2025-10-29

**Authors:** Xinyan Zhou, Jinyan Huang, Yu Zhu, Yufei Wei, Liyuan Liao, Jiaoming Li, Yi He, Shangdong Yang

**Affiliations:** 1 Guangxi Key Laboratory of Agro–environment and Agro–products Safety, National Demonstration Center for Experimental Plant Science Education, College of Agriculture, Guangxi University, Nanning, 530004 Guangxi P.R. China; 2 Horticultural Research Institute, Guangxi Academy of Agricultural Sciences, Nanning, 530007 Guangxi P.R. China

**Keywords:** Watermelon (*Citrullus lanatus* (Thunb.) matsum.), *Fusarium* wilt, Roots, Endophytic microbes, Metabolites

## Abstract

**Background:**

The structure of endophytic microbial communities and metabolic functions differ significantly among plant varieties with different resistance levels. Currently, there is a lack of research articles that combine microbiomics and metabolomics to explore the mechanism of resistance to wilt disease in watermelon. To seek out the antagonistic microorganisms and metabolites against watermelon wilt from different watermelon varieties, we investigated the characteristics of endophytic microbial communities, metabolic features and functions in the roots of wilt–resistant (RW) and susceptible (SW) watermelon varieties.

**Results:**

The results suggested that significant differences of endophytic microbial communities and metabolites could be found in the roots between RW and SW. Meanwhile, the endophytic bacterial genera such as *Chryseobacterium*, *Pseudomonas*, *Delftia*, *Lechevalieria*, *unclassified_f__Methylophilaceae*, *Tahibacter*, and the endophytic fungal genera, *unclassified_p__Basidiomycota*, *Neocosmospora*, *unclassified_f__Lasiosphaeriaceae*, *Edenia* were the unique dominant bacterial and fungal genera in the roots of RW, respectively. Additionally, the differential metabolites, including Galactinol, Sucrose, Stachyose, Coniferyl Aldehyde, Coniferin, 5–Hydroxyconiferyl alcohol, 4–Coumaryl alcohol, 3–Hydroxybenzoic Acid, and the metabolic pathways including Galactose metabolism, Phenylpropanoid biosynthesis, Phenylalanine, tyrosine and tryptophan biosynthesis significantly upregulated in wilt resistant watermelon varieties.

**Conclusion:**

This study systematically reveals, for the first time, the synergistic defense mechanisms between root endophytic microbiome and metabolome during *Fusarium* wilt resistance formation in watermelon. Significantly, we have identified potential functional microorganisms, key metabolites, and critical pathways that actively contribute to these defense mechanisms. However, the specific functions of these potential antagonistic microorganisms and metabolites still need further validation. These findings provide a novel perspective for crop disease resistance research, transcending the limitations of traditional single-factor analytical paradigms, while establishing a methodological foundation for developing multi-omics integrated approaches in crop disease resistance regulation strategies.

**Supplementary Information:**

The online version contains supplementary material available at 10.1186/s12870-025-07488-5.

## Background

Watermelon is globally cultivated in over 96 countries/regions and is one of the most widely planted horticultural crops. In 2023, the annual production of watermelon in the world was approximately 105 million tons (FAOSTAT, http://faostat.fao.org). However, watermelon diseases are also gradually becoming severe, and *Fusarium* wilt is one of the most serious diseases occurring during the growth period of watermelon [1]. Watermelon wilt is a soil–borne disease caused by *Fusarium oxysporum* [2], which often invades from watermelon roots and extends along the vascular bundles towards the aboveground parts. This invasion results in cell membrane rupture, vascular bundle blockage, and ultimately leads to wilting and plant death [3, 4]. Particularly, a 20%–30% yield reduction with severe cases reaching 50–60% or even total crop failure, can significantly affect watermelon production [5]. While conventional strategies such as chemical fungicides and crop rotation have been employed to manage *Fusarium* wilt, their efficacy remains limited due to pathogen persistence in soil and emerging fungicide resistance [6]. Against this background, the use of resistant watermelon varieties represents an environmentally friendly and effective strategy for the control of Fusarium wilt in watermelon [7].

Recently, plant microbiomes have gained considerable attention due to their positive effects on host plant health and adaptation [8, 9]. Plant-associated microbiomes can influence various plant traits, including growth and tolerance to abiotic stresses [10], and are also closely related to host disease resistance or susceptibility [11]. Endophytic microorganisms are an important component of the plant microbiome. Endophytic microorganisms inhabit healthy plants’ intercellular spaces or intracellular compartments, thriving in a relatively stable environment and allowing for long–term colonization within plants with a stabilizing influence [12]. Meanwhile, based on coevolution with their host plants, endophytic microorganisms have established a mutually beneficial and regulated symbiotic relationship, fostering a harmonious coexistence [13]. For example, the interaction between *Clethra barbinervis* and endophytic fungi in the roots enhances plant tolerance to heavy metals and improves the absorption of mineral nutrients [14]; Dark septate endophytes (DSEs) play a significant role in promoting the growth of rice (*Oryza sativa*), enhancing mineral absorption, preventing biological diseases, and increasing the resistance of rice to stress [15]. Meanwhile, previous studies indicated that plant resistance plays a crucial role in changing the endophytic microbial compositions. Kiani et al. [16] found that significant differences in endophytic bacterial quantity could be detected among wheat varieties resistant and susceptible to rust disease; Tian et al. [17] also found that different leaf–associated bacterial compositions could be detected in various corn varieties with resistance and susceptibility to Northern leaf blight. Additionally, Majumdar et al. [18] discovered that maize varieties resistant to *Aspergillus flavus* have more diverse and stable endophytic communities compared to susceptible varieties.

Plant resistance is also closely related to its metabolomic characteristics. Plants can produce thousands of unique metabolites that serve to attract pollinators, provide protection against environmental stresses, and combat microbial pathogens [19]. Metabolites such as flavonoids, phenylpropanoids, and coumarins play critical roles in plant defense mechanisms [20, 21]. Meanwhile, metabolomic profiles are also regulated by plant resistance traits. Compared to susceptible varieties, resistant varieties often exhibit significantly higher levels of phenylpropanoids, flavonoids, coumarins, and carbohydrate metabolites [22–24]. Additionally, endophytic microorganisms are closely associated with the metabolic characteristics within plants. Beneficial endophytic microbes can produce a variety of advantageous secondary metabolites, thereby regulating numerous metabolic and physiological activities in plants [25], activating the plant’s defense mechanisms, and inducing host plants to respond to adverse environmental conditions [26–28]. Moreover, plants can influence the assembly of their microbiome by synthesizing various metabolites. These metabolites not only act as chemical signals mediating plant-microbe interactions but also selectively promote the colonization of beneficial microorganisms while inhibiting the growth of potential pathogens [29, 30].

Given the severe threat that *Fusarium* wilt poses to the global watermelon industry and the limitations of traditional control strategies, uncovering the intrinsic disease resistance mechanisms mediated by plant-microbe interactions in watermelon has become an urgent scientific challenge. Although previous studies have recognized the roles of plant microbiomes and metabolomes in disease resistance, the synergistic mechanisms between these two factors—particularly the interactive networks between root microecology and host metabolic regulation—remain largely unexplored. In this study, we innovatively integrate microbiome and metabolome profiling technologies, focusing on the root microenvironment of *Fusarium* wilt-resistant and -susceptible watermelon cultivars. Our aim is to elucidate the resistance formation mechanisms from a multidimensional “microbe-metabolite-host” interaction perspective, while identifying potential antagonistic microorganisms, key metabolites, and critical pathways contributing to resistance.

## Materials and methods

### Field site description and experimental designs

The experiment was carried out at the experimental base of Suxu town (108° 6′11″ E, 22° 28′ 28″ N), Nanning city, Guangxi Zhuang Autonomous Region, southwest China. The wilt–resistant watermelon varieties are as follows: Guixuan103, Guixuan104 and Guixuan118; abbreviated RW group. By contrast, the wilt–susceptible watermelon varieties are as follows: Guixiao Hei, Guixiao Mei and Guixiao Xiu; abbreviated SW group. They were all tested and provided by watermelon breeding team of the Horticultural Research Institute, Guangxi Academy of Agricultural Sciences, China. All varieties were concurrently grown seedlings, transplanted, and were all under the same management. Wilt–resistant and susceptible watermelon root samples were randomly collected from each variety on May 26, 2023. Root samples from every watermelon variety were collected randomly according to the method described by Yang et al. [31]. Briefly, three watermelon plants with consistent growth from each variety were randomly selected. Using a sterilized shovel, the soil within a 25 cm radius around the base of the plant was loosened, and then the entire watermelon plant was pulled out by hand, holding the base of the stem. The large soil clumps and soil attached to the roots were shaken off, and the roots were carefully cut off using sterilized scissors, placed in sterile self-sealing bags, labeled, and brought back to the laboratory. The soil and debris attached to the roots were gently brushed off with a soft-bristled brush, and the root samples were rinsed with sterile water. After being blotted dry with sterile paper, the roots were transferred to a 2% (v/v) NaClO solution for thorough soaking for 15 min. After five rinses with sterile water, the roots were transferred to a 70% (v/v) ethanol solution for soaking for 5 min [32]. Subsequently, five more rinses with sterile water were performed, and the roots were blotted dry with sterile paper. The watermelon roots were cut into approximately 1 cm segments using sterile scissors. For each variety within both groups, three biological replicates were collected, resulting in 9 samples (1.0 g each) from each group placed into sterile sealable bags for endophytic microbial community analysis. Additionally, for root metabolome analysis, 6 biological replicates were collected from each variety, leading to a total of 18 samples (5.0 g each) per group (3 varieties×6 replicates).

### Test methods

Sample grinding and DNA extraction: 0.5 g of root samples were weighed and placed into a sterilized grinding bowl, to which liquid nitrogen was added. The samples were then ground using a pestle until a fine powder was obtained. The powder was subsequently transferred to a 50 mL sterile centrifuge tube for DNA extraction. Total DNA extraction from watermelon root samples was performed using the Fast DNA^®^ Spin Kit for Soil (MP Biomedicals, Thomas Irvine, CA, USA) according to the manufacturer’s instructions. The extracted DNA was examined on 1% agarose gel to verify extraction quality, followed by gel electrophoresis. The concentration and purity of the extracted DNA were determined using a NanoDrop 2000 spectrophotometer (Thermo Fisher Scientific, Waltham, MA, USA) [33]. Following extraction, the DNA was stored at − 20 °C for further processing.

PCR amplification: TransStart Fastpfu DNA Polymerase was used for Polymerase Chain Reaction (PCR) amplification. The bacterial 16S rRNA gene was amplified using the primer pair 799 F (5’–AACMGGATTAGATACCCKG–3’) and 1193R (5’–ACGTCATCCCCACCTTCC–3’) [34]. For the amplification of fungal ITS regions, the primer pair ITS1F (5’–CTTGGTCATTTAGAGGAAGTAA–3’) and ITS2R (5’–GCTGCGTTCTTCATCGATGC–3’) was employed [35]. The PCR protocol involved an initial denaturation at 95 °C for 3 min, followed by denaturation cycles (16 S rRNA gene: 27 cycles; ITS gene: 35 cycles) at 95 °C for 30 s, annealing at 55 °C for 30 s, and extension at 72 °C for 72 s. This was concluded with a final extension at 72 °C for 10 min and termination at 4 °C.

Subsequently, the PCR products were recovered and purified using the AxyPrep DNA Gel Extraction Kit (Axygen Biosciences, Union City, CA, USA) after detection through 2% agarose gel electrophoresis. The purified products were further confirmed by 2% agarose gel electrophoresis, and their quantification was performed using the Quantus Fluorometer (Promega, Madison, WI, USA). Purified amplicons were pooled in equimolar amounts and were then paired–end sequenced on Illumina MiSeqPE300 (bacteria) and MiSeqPE250 (fungi) platforms (Illumina, San Diego, USA) by Majorbio Bio–Pharm Technology Co. Ltd. (Shanghai, China) according to standard protocols.

The raw data for the soil bacterial and fungal sequences were deposited in the NCBI Sequence Read Archive database under the accession numbers PRJNA1080270 and PRJNA1080342, respectively. (Note: In this study, both the resistant watermelon group (RW) and susceptible watermelon group (SW) consisted of three distinct varieties: Guixuan103, Guixuan104, and Guixuan118 for RW, and Guixiao Hei, Guixiao Mei, and Guixiao Xiu for SW. Each variety included 3 biological replicates, resulting in a total of 18 samples analyzed in this work. Other varieties’ samples were also sequenced, uploaded to the public database, and reserved for subsequent studies, so they were not included in the current analysis.)

Raw FASTQ files from the 18 samples (as described in the Note) were demultiplexed using an in-house Perl script, quality-filtered using fastp version 0.19.6, and merged using FLASH version 1.2.7. The following criteria were used: (i) the reads were truncated at any site with an average quality score of < 20 over a 50-bp sliding window; truncated reads shorter than 50 bp were discarded, as were reads containing ambiguous characters; (ii) only overlapping sequences longer than 10 bp were merged based on their overlapping, with a maximum mismatch ratio of 0.2 (i.e., 20%) in the overlapping region; reads that could not be merged were discarded; (iii) samples were distinguished using barcodes and primers, with sequence direction adjusted to allow exact barcode matching and two nucleotide mismatches in primer matching. Processed sequences were then clustered into operational taxonomic units (OTUs) using UPARSE 7.1 at a 97% sequence similarity level. The most abundant sequence of each OTU was selected as the representative sequence. Taxonomic annotation of each OTU representative sequence was performed using RDP Classifier version 2.11 against the 16 S rRNA gene database (Release138 http://www.arb-silva.de) and the Fungal UNITE database (Release 8.0 http://unite.ut.ee/index.php) with a confidence threshold of 0.7 [36].

Untargeted metabolomic assays and analysis [37, 38]: A 50 mg sample was accurately transferred to a 2.0 mL centrifuge tube, a 6 mm diameter abrasive bead was added; methanol and ultrapure water were mixed in a 4:1 ratio, and then 400 µL of this solution was added to the sample for extraction. The frozen tissue grinder was ground for 6 min (–10℃, 50 Hz). After vortex mixing, the low–temperature ultrasonic extractor was set to 5℃, and ultrasonic extraction was performed at 40 kHz for 30 min. After extraction was completed, the sample was placed in a freezer at − 20℃ for 30 min and centrifuged at 4℃, 13,000 g for 15 min. After centrifugation, the supernatant was absorbed and dried with nitrogen. A mixture of acetonitrile and water at 1:1 was used as the compound solution; 120 µL was absorbed and redissolved, and then vortex mixing was performed. After low–temperature ultrasonic extraction, the sample was centrifuged at 4℃ for 10 min, and the supernatant was absorbed and transferred to an injection vial with intubation. Ultrahigh–performance liquid chromatography tandem Fourier transform mass spectrometry was performed on a UHPLC–Q Exactive HF–X system (Thermo Fisher Scientific, USA) system for LC‒MS detection. In addition, 20 µL of supernatant was removed from each sample and used as a quality control sample. The chromatographic conditions were as follows: the chromatography column used was an ACQUITY UPLC HSS T3 (100 mm × 2.1 mm, i.d. 1.8 μm; Waters, Milford, USA); mobile phase A was 95% water + 5% acetonitrile (including 0.1% formic acid), and mobile phase B was 47.5% acetonitrile + 47.5% isopropanol + 5% water (including 0.1% formic acid), and mobile phase B was 47.5% acetonitrile + 47.5% isopropanol + 5% water (include 0.1% formic acid). The flow rate was set to 0.40 mL/min, the injection volume was 3 µL, and the column temperature was 40℃. The Majorbio cloud platform (https://cloud.majorbio.com) was used for multivariate analysis.

### Statistical analysis

The data were statistically analyzed using Excel 2019 and Statistical Product and Service Solutions (SPSS) Statistics 21, and the results are shown as the means with their standard deviations (mean ± SD). Based on OTU information, alpha diversity indices, including observed OTUs, Shannon diversity index, and Chao1 richness index, were calculated using Mothur (version v.1.30.2 https://mothur.org/wiki/calculators/). Beta diversity was assessed via principal coordinates analysis (PCoA) based on Bray–Curtis dissimilarity matrices calculated from the OTU abundance data. The Bray–Curtis distances were computed using the vegdist function in the vegan package (version 2.6–4.6) in R (version 3.3.1). Based on these distance, PCoA was performed using the pcoa function in the ape package (version 5.7-1.7) [39], and plots were visualized with the ggplot2 package (version 3.4.4) [40]. Partial least squares discriminant analysis (PLS-DA) was conducted to identify variables that best distinguished among predefined groups using the mixOmics package (version 6.26.0) in R [41]. Linear discriminant analysis (LDA) effect size (LEfSe; http://huttenhower.sph.harvard.edu/LEfSe) was employed to identify bacterial and fungal taxa (from phylum to genus level) with significant abundance differences between the RW and SW groups, using criteria of LDA score >3.0 and *p* < 0.05. Bacterial community functions were predicted using FAPROTAX software (version 1.2.1), while fungal community functions were predicted using FUNGuild (http://www.funguild.org/).The co–generation network was visualized using Gephi 0.10.2 [42]. Metabolic group data were analyzed using KEGG (www.kegg.jp/kegg/kegg1.html) developed by Kanehisa Laboratories [43]. The selection of significantly different metabolites was determined based on the variable weight values (VIP) and Student’s t-test P-values obtained from the PLS-DA model; metabolites with VIP >1 and *p* < 0.05 were classified as significantly different metabolites. KEGG pathway enrichment analysis was performed to identify significantly enriched metabolic pathways among the differentially abundant metabolites/genes. Input data consisted of the list of differential metabolites with their corresponding KEGG identifiers. Enrichment analysis was conducted using the clusterProfiler package (version 4.6.0) in R [44], which implements statistical methods to assess over-representation of KEGG pathways. Adjusted p-values < 0.05 were considered significant. The enrichment results were visualized using the ggplot2 package (version 3.4.4) in R. Online data analysis was performed using the free online Majorbio Cloud Platform (http://www.majorbio.com) of the Majorbio Bio–Pharm Technology Co., Ltd. (Shanghai, China).

## Results

### Root endophytic bacterial and fungal alpha and beta diversities

**Fig. 1:**
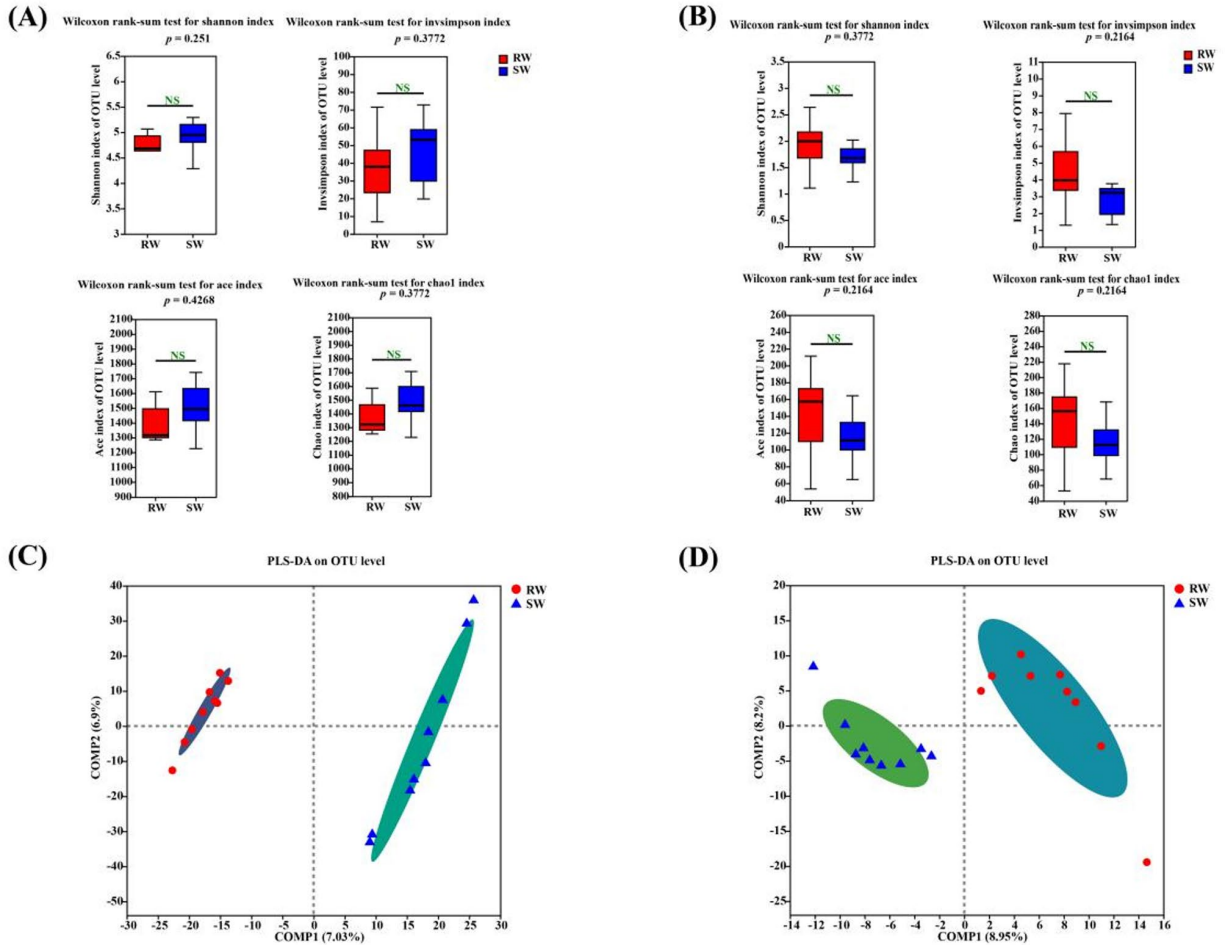
Alpha diversities of endophytic bacterial (**A**) and fungal (**B**) communities: PLS–DA score plot of endophytic bacterial (**C**) and fungal (**D**) communities

As shown in Fig. 1A, the Shannon, Invsimpson, Ace, and Chao1 indices of endophytic bacteria in the roots did not differ significantly between wilt-resistant (RW) and wilt-sensitive (SW) watermelon varieties, indicating similar diversity and abundance in both groups. Similarly, these diversity indices for endophytic fungi (Fig. 1B) showed the same pattern, with no significant differences between RW and SW, suggesting comparable fungal diversity and abundance in the two groups.

However, based on the results of Partial Least Squares Discriminant Analysis (PLS–DA), the endophytic bacterial communities in the roots of RW and SW could be distinctly differentiated and grouped into two clusters (Fig. 1C). Also, the endophytic fungal communities in the roots of RW and SW could be separately grouped into two clusters (Fig. 1D). All above results suggested that significant difference of endophytic bacterial and fungal compositions could be detected in the roots of RW and SW, respectively.

### Root endophytic bacterial compositions

**Fig. 2:**
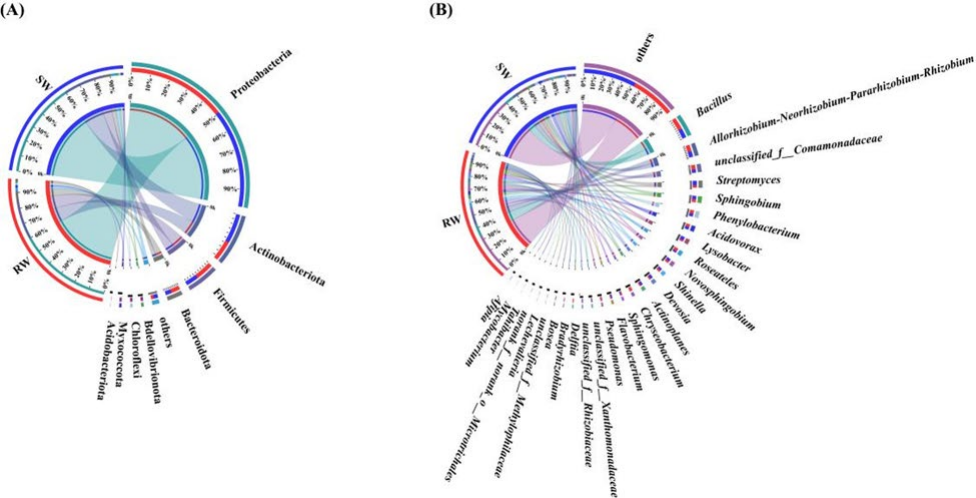
Endophytic bacterial compositions at the phylum (**A**) and genus (**B**) levels

As shown in Fig. 2A, the numbers of dominant endophytic bacterial phyla (relative abundance > 1%) in the roots of RW and SW were 7 and 6, respectively. The common dominant endophytic bacterial phyla in the roots of both RW and SW included Proteobacteria (61.00–60.94%), Actinobacteriota (13.03–18.55%), Firmicutes (11.87–8.23%), Bacteroidota (6.33–4.18%), Myxococcota (1.49–1.33%), Chloroflexi (1.44–1.45%), and Bdellovibrionota (1.42–1.53%). Compared with SW, Acidobacteriota (1.11%) was uniquely dominant in the roots of RW.

Additionally, the numbers of dominant endophytic bacterial genera (relative abundances > 1%) in the roots of RW and SW were 24 and 22, respectively (Fig. 2B).

Firstly, *Bacillus* (10.09–7.61%), *Allorhizobium-Neorhizobium-Pararhizobium-Rhizobium* (5.84–4.94%), *unclassified_f__Comamonadaceae* (4.22–3.24%), *Roseateles* (3.39–1.83%), *Phenylobacterium* (3.37–2.99%), *Sphingobium* (2.97–1.51%), *Acidovorax* (2.89–2.88%), *Novosphingobium* (2.43–2.71%), *Streptomyces* (2.38–4.40%), *Shinella* (2.33–2.35%), *Devosia* (1.82–2.84%), *Sphingomonas* (1.82–1.51%), *Lysobacter* (1.79–3.56%), *Actinoplanes* (1.78–2.55%), *Flavobacterium* (1.42–1.89%), *Bradyrhizobium* (1.18–1.04%), *unclassified_f__Xanthomonadaceae* (1.11–1.55%), and *Bosea* (1.08–1.12%) were the common dominant endophytic bacterial genera in the roots of RW and SW.

Meanwhile, *unclassified_f__Rhizobiaceae* (1.69%), *norank_f__norank_o__Microtrichales* (1.25%), *Mycobacterium* (1.10%), *Afipia* (1.02%) were the unique dominant endophytic bacterial genera in the roots of SW. In contrast, *Chryseobacterium* (3.70%), *Pseudomonas* (2.44%), *Delftia* (2.43%), *Lechevalieria* (1.43%), *unclassified_f__Methylophilaceae* (1.15%), *Tahibacter* (1.11%) were the unique dominant endophytic bacterial genera in the roots of RW.

### Root endophytic fungal compositions

**Fig. 3:**
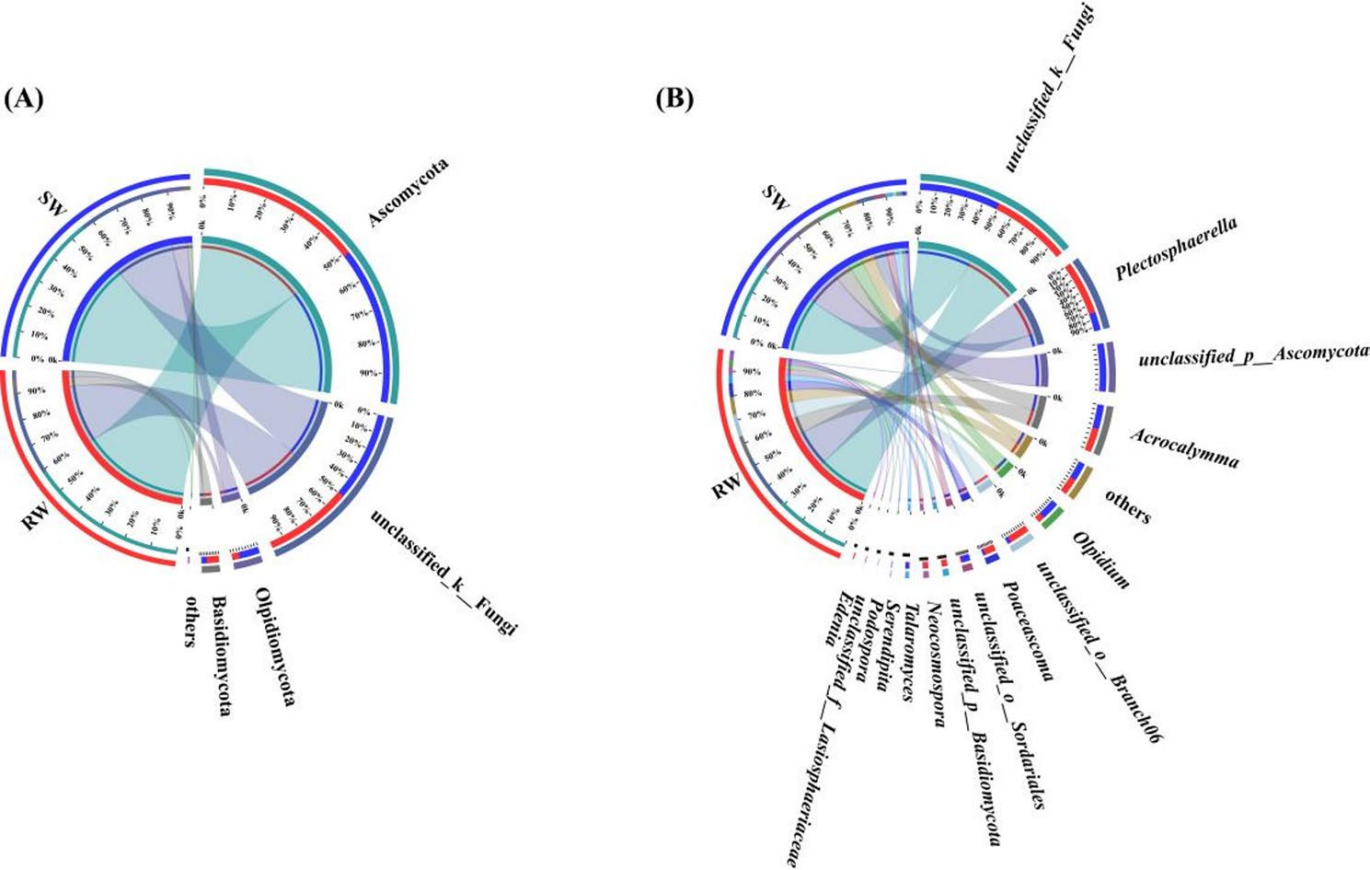
Endophytic fungal compositions at the phylum (**A**) and genus (**B**) levels

As shown in Fig. 3A, the common dominant endophytic fungal phyla in the roots of RW and SW were Ascomycota (59.65–56.54%), unclassified_k__Fungi (31.69–32.40%), Basidiomycota (5.30–2.32%) and Olpidiomycota (2.98–8.26%).

As shown in Fig. 3B, *unclassified_k__Fungi* (31.69–32.40%), *Plectosphaerella* (22.30–7.56%), *Acrocalymma* (10.12–10.20%), *unclassified_o__Branch06* (9.42–1.58%), *Poaceascoma* (5.61–1.19%), and *Olpidium* (2.98–8.26%) were the common dominant endophytic fungal genera in the roots of RW and SW.

Meanwhile, *unclassified_p__Ascomycota* (20.14%), *unclassified_o__Sordariales* (4.58%), *Talaromyces* (3.02%), *Podospora* (1.35%), *Serendipita* (1.23%) were the unique dominant endophytic fungal genera in the roots of SW. By contrast, *unclassified_p__Basidiomycota* (3.70%), *Neocosmospora* (3.26%), *unclassified_f__Lasiosphaeriaceae* (1.02%), *Edenia* (1.02%) were the unique dominant endophytic fungal genera in the roots of RW.

### The LEfSe analysis of root endophytic bacterial and fungal communities

LEfSe analysis was also employed to identify taxonomic groups of bacterial and fungal communities from phylum to genus level with Linear Discriminant Analysis (LDA) scores exceeding 3.0 in the roots of RW and SW.

**Fig. 4:**
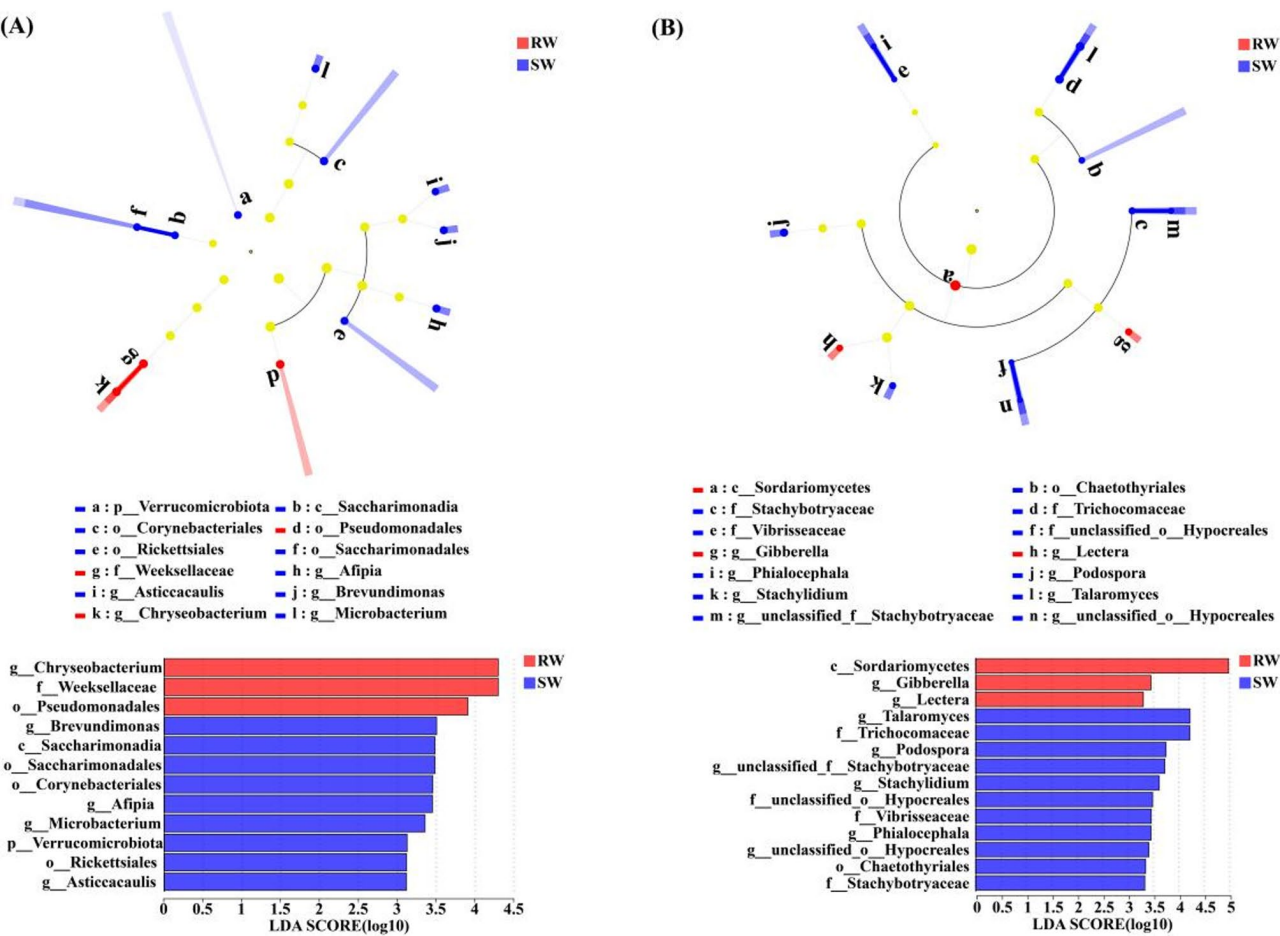
LEfSe analysis of significant abundances of endophytic bacteria (**A**) and fungi (**B**) in the roots between RW and SW

As shown in Fig. 4A, the endophytic bacterial communities in the roots between RW and SW revealed significant differences in 12 taxa. At the genus level, *Chryseobacterium* was identified as a biomarker in the roots of RW. On the contrary, *Brevundimonas*, *Afipia*, *Microbacterium*, and *Asticcacaulis* were identified as biomarkers in the roots of SW.

Also, the endophytic fungal communities in the roots between RW and SW showed significant differences in 14 taxa (Fig. 4B). At the genus level, *Gibberella* and *Lectera* were identified as biomarkers in the roots of RW. *Talaromyces*, *Podospora*, *unclassified_f__Stachybotryaceae*, *Stachylidium*, *Phialocephala*, and *unclassified_o__Hypocreales* were identified as biomarkers in the roots of SW.

### Endophytic bacterial and fungal community co–generation network structure

**Fig. 5:**
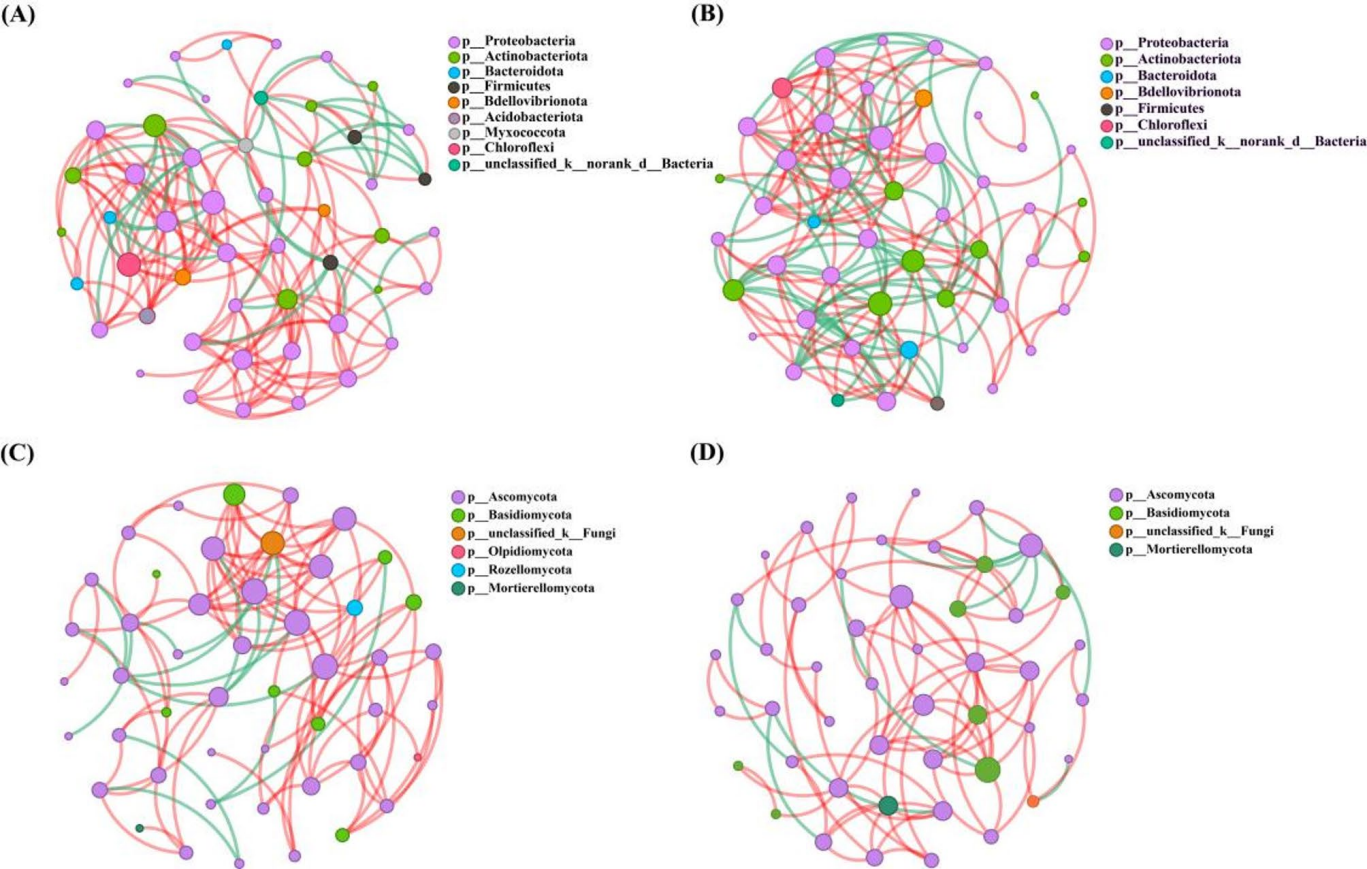
Co–generation network of the bacteria of RW (**A**) and SW (**B**); Co–generation network of the fungi of RW (**C**) and SW (**D**); Each node represents a single genus, different colors represent differentand phylum, the color of edges indicates the type of interaction. Red, positive; Green, negative

The Spearman rank correlation coefficient was calculated based on the top 50 bacterial and fungal genera with the highest relative abundances. Meanwhile, a unifactorial correlation network analysis was also conducted to examine the interrelationships among dominant species. The results showed that Proteobacteria was the dominant bacterial phylum among the network interactions in the roots of RW and SW, accounting for 58% and 67.35%, respectively. Particularly, it was 77.71% positive and 22.29% negative connections in the roots of RW (Fig. 5A); In contrast, it was 58.29% positive and 41.71% negative connections in the roots of SW (Fig. 5B). Moreover, Ascomycota was the dominant fungal phylum among the network interactions in the roots of RW and SW, constituting 75% and 80.85%, respectively. And it was 86.36% positive and 13.64% negative connections in the roots of RW (Fig. 5C); By contrast, it was 84.78% positive and 15.22% negative connections in the roots of SW (Fig. 5D).

### Metabolite in the roots of wilt–resistant and susceptible watermelon varieties

**Fig. 6:**
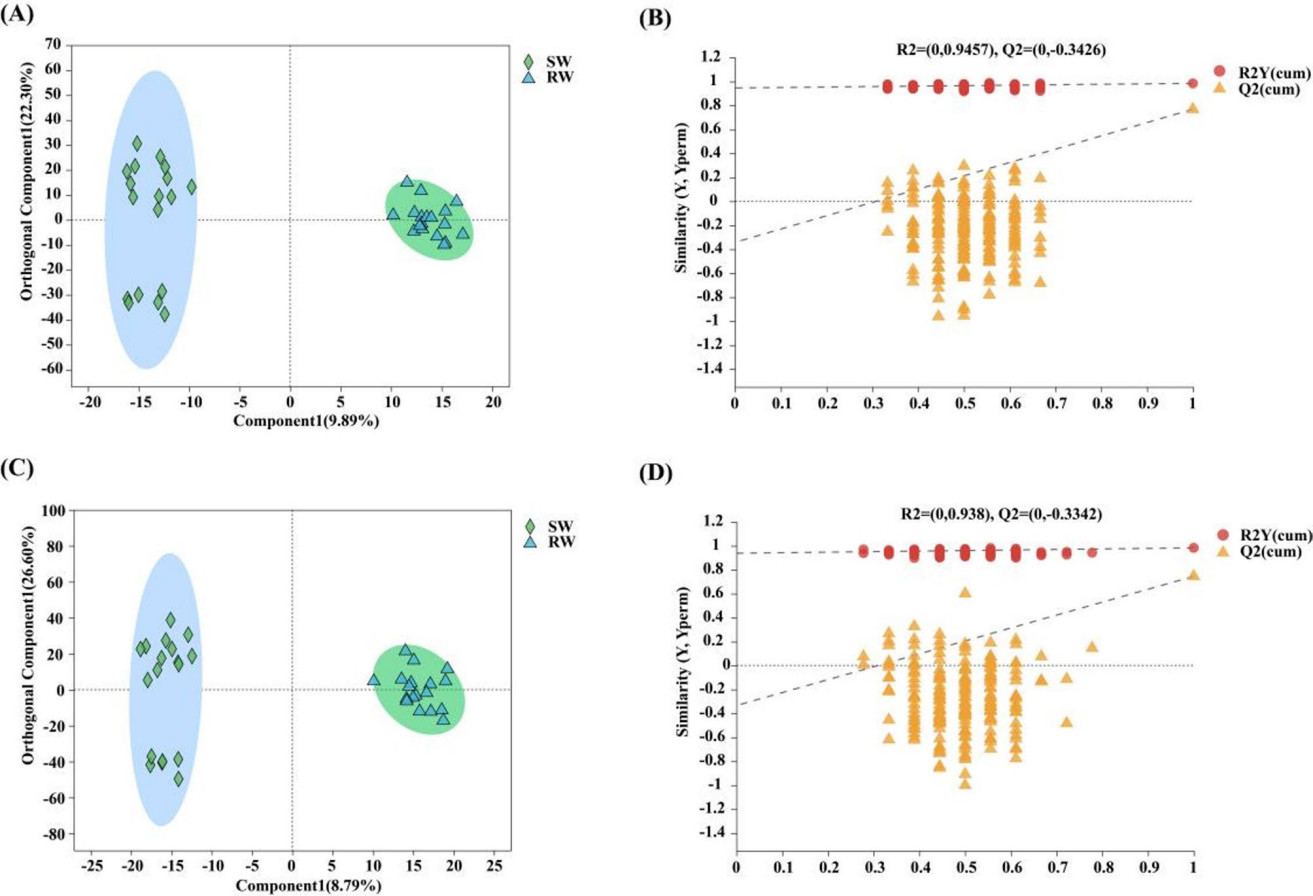
Orthogonal partial least squares discriminant analysis (OPLS–DA) plot of metabolites in the roots of RW and SW. **A** positive mode ionization; **B** permutation test of OPLS–DA in positive mode ionization; **C** negative mode ionization; **D** permutation test of OPLS–DA in negative mode ionization

Firstly, the OPLS–DA analysis showed that in the positive ion mode, the two principal components PC1 and PC2 explained 9.89% and 22.30% of the variance, respectively (Fig. 6A); in the negative ion mode, PC1 and PC2 explained 8.79% and 26.60% of the variance, respectively (Fig. 6C). The metabolites in the roots of RW and SW exhibited good intra–group cohesion and repeatability in both ion modes, and were clearly separated into distinct clusters, indicating significant differences in root metabolite compositions between RW and SW. To ensure the reliability of the results, a permutation test analysis was conducted on the OPLS–DA model for 200 iterations. R2Y and Q2 were utilized to assess the fitting and predictive capabilities of the OPLS–DA model, where larger cumulative values of R2Y and Q2 indicate greater stability and reliability of the model. In the positive ion mode, the cumulative values were 0.984 for R2Y and 0.768 for Q2 (Fig. 6B); in the negative ion mode, the cumulative values were 0.983 for R2Y and 0.745 for Q2 (Fig. 6D). Throughout the 200 permutation tests, randomly generated R2 and Q2 values were consistently much smaller than those of the original values, and exhibited significant negative slope in the linear regression. Meanwhile, all intercepts of the Q2 regression lines were less than 0, it also indicated that there was no overfitting in the OPLS–DA model. Moreover, the variable importance in projection (VIP) values were used for the subsequent selection of metabolites.

**Fig. 7:**
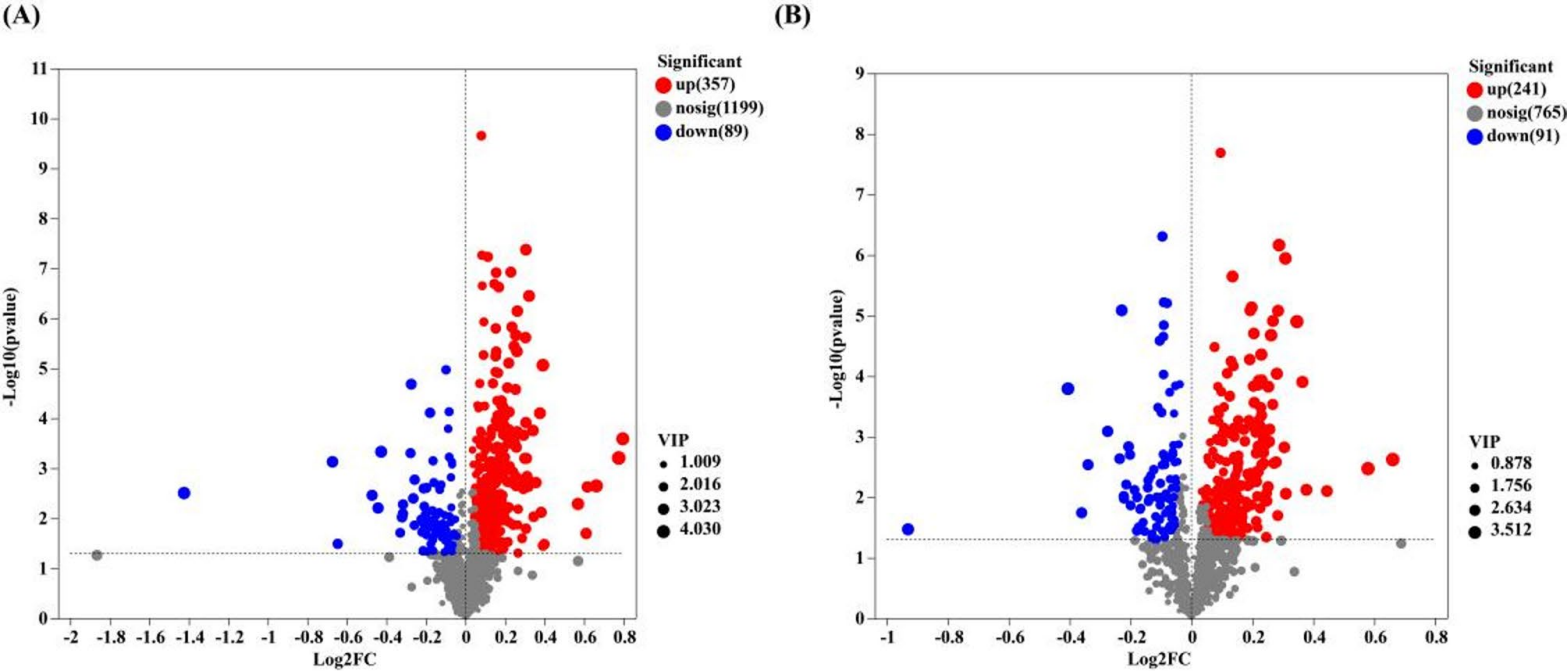
Different metabolites in the roots between RW and SW. **A** Volcano plots of differential metabolites in the roots between RW and SW in positive ion mode; **B** Volcano plots of differential metabolites in the roots between RW and SW in negative ion mode; Red indicates upregulation, blue indicates downregulation, and gray indicates non–significant differences

To filter out significantly different metabolites in the roots between RW and SW, OPLS–DA VIP > 1 and *p* < 0.05 were used as evaluation criteria for screening. Based on the screening and statistical analysis results, a total of 778 differential metabolites were identified in this experiment. In the positive ion mode, 446 differential metabolites were selected, with 357 upregulated in RW (Fig. 7A). In the negative ion mode, 332 differential metabolites were identified, with 241 upregulated in RW (Fig. 7B).

**Fig. 8:**
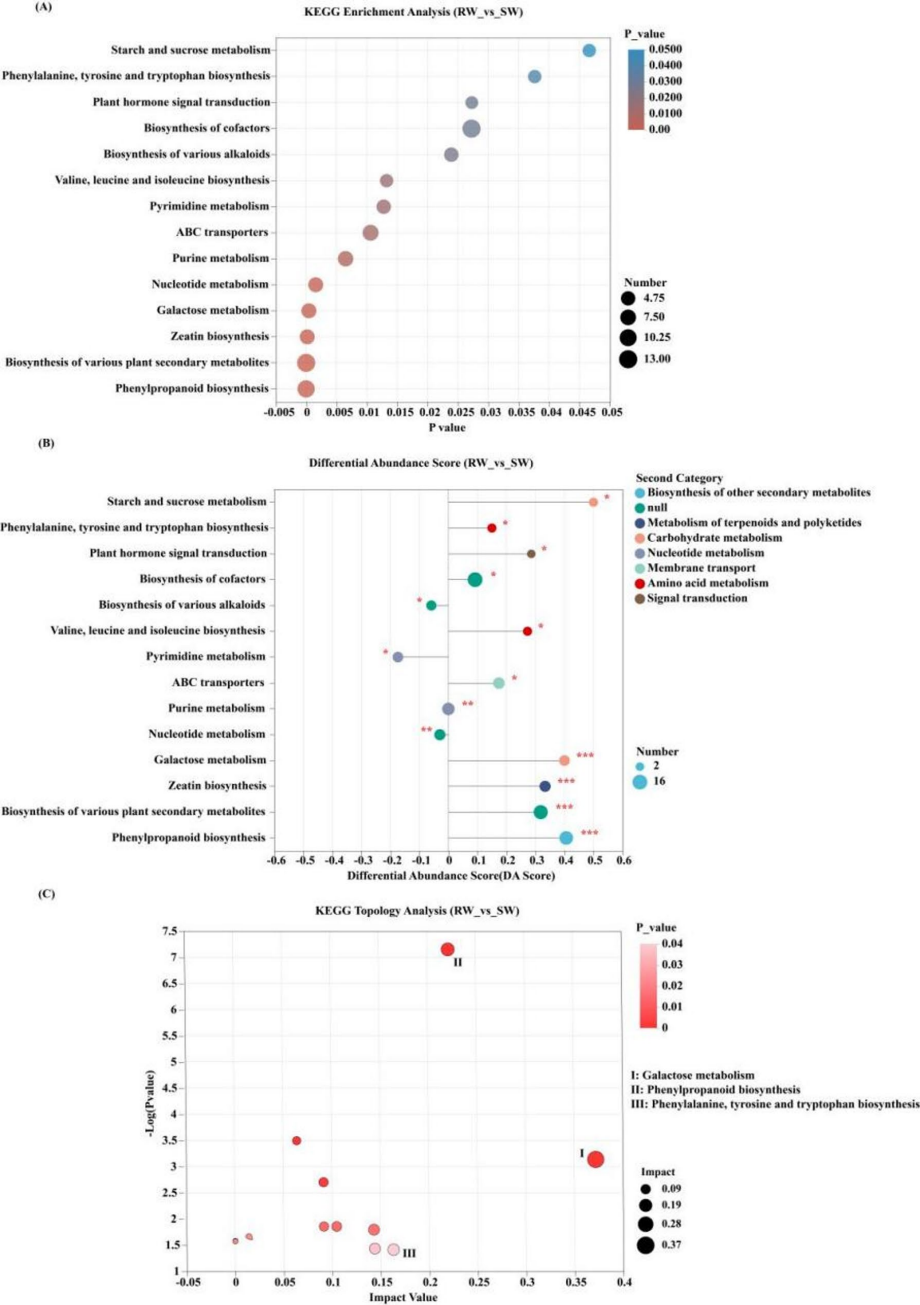
Enrichment analysis of KEGG pathway for differential metabolites (**A**) Bubble diagram for KEGG enrichment analysis. **B** KEGG pathway differential abundance score map. **C** KEGG topology analysis of bubble diagrams

A total of 778 differential metabolites were annotated using the KEGG PATHWAY database. Enrichment analysis of the differential metabolites was conducted using the hypergeometric distribution algorithm to identify significantly enriched pathways. The results showed that 80 differential metabolites were significantly enriched in 14 metabolic pathways (The differential metabolites enriched in the KEGG metabolic pathway (*p <* 0.05) are shown in Supplementary material 1), leading to notable changes in their expression levels (Fig. 8A). In the roots of RW, ten metabolic pathways showed significantly elevated expression levels compared to SW, including Starch and sucrose metabolism, Phenylalanine, tyrosine and tryptophan biosynthesis, Plant hormone signal transduction, Biosynthesis of cofactors, Valine, leucine and isoleucine biosynthesis, ABC transporters, Galactose metabolism, Zeatin biosynthesis, Biosynthesis of various plant secondary metabolites, and Phenylpropanoid biosynthesis; Conversely, the roots of SW exhibited higher expression levels in three pathways relative to RW: Biosynthesis of various alkaloids, Pyrimidine metabolism, and Nucleotide metabolism (Fig. 8B). Furthermore, to assess the impact factors of metabolic pathways, KEGG topological analysis was simultaneously conducted. The results showed that the top three pathways with the highest impact factors were Galactose metabolism, Phenylpropanoid biosynthesis, Phenylalanine, and tyrosine and tryptophan biosynthesis (Fig. 8C).

### Correlation analysis of metabolites and endophytic microbes in the roots between wilt–resistant and susceptible watermelon varieties

Based on the top 30 ranked root metabolites, the Spearman correlation analysis was performed on the dominant bacterial and fungal genera in root between resistant (RW) and susceptible (SW) watermelon varieties. The results suggested that a strong correlation of metabolites and the dominant bacterial and fungal genera in the roots systems between RW and SW.

In terms of bacterial genera, a significant negative correlation was observed between *Flavobacterium* and Heme, Lactose, Sucrose; However, *Devosia* showed a significant positive correlation with Glutathione, oxidized, and Cucurbitacin I; Also, *Sphingobium* exhibited a significant positive correlation with Cucurbitacin I and a significant negative correlation with Trans–Cinnamaldehyde, DADP, Cinnamaldehyde; Meanwhile, *Novosphingobium* showed a significant negative correlation with Trans–Cinnamaldehyde, DADP, Cinnamaldehyde, Adenosine 5’–Monophosphate; And *Actinoplanes* showed a significant positive correlation with Aldosterone 18–glucuronide; Moreovre, *unclassified_f__Comamonadaceae* exhibited a significant negative correlation with Uridine–5’–monophosphate, 3’,5’–Cyclic GMP, Adenosine 5’–Monophosphate, Glutathione and oxidized.

**Fig. 9:**
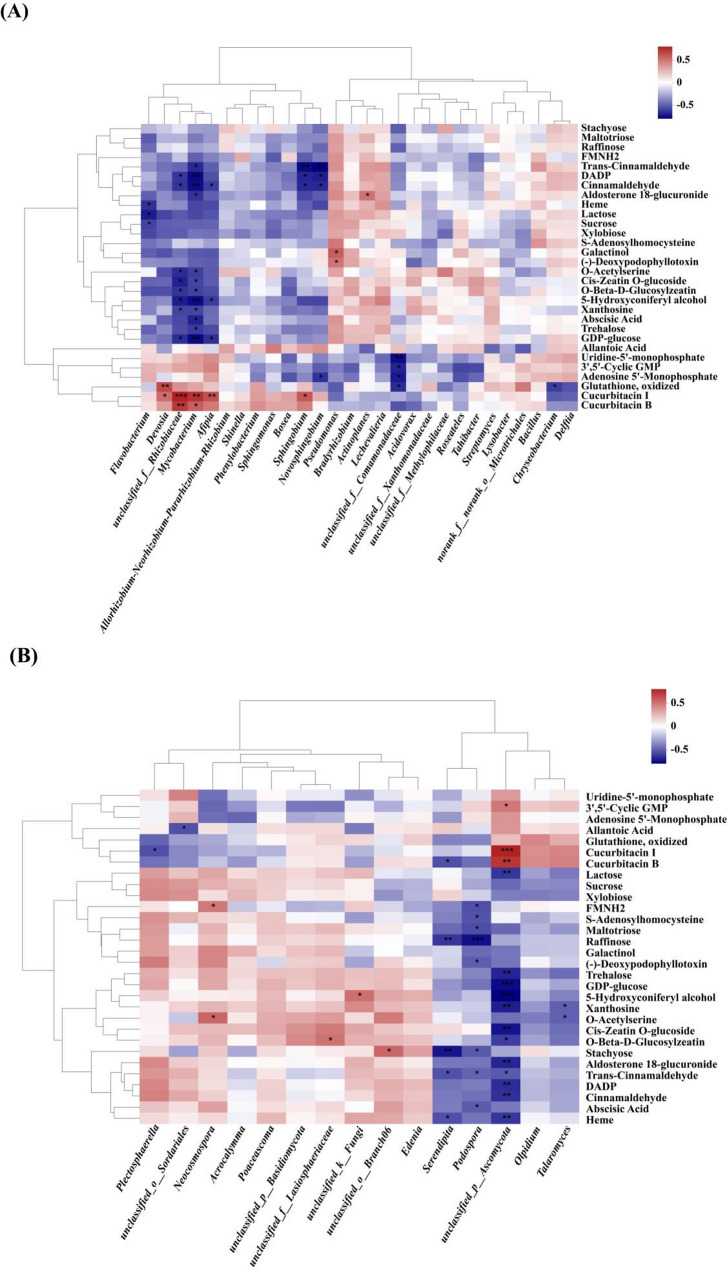
Correlations of metabolites and endophytic microbial communities in the roots between the wilt–resistant and susceptible watermelon varieties * 0.01 < *p* < 0.05, ** 0.001 < *p* < 0.01, *** *p <* 0.001. The clusters at the top and left of the figures show the results of metabolite with Euclidean distance–based hierarchical clustering of bacterial and fungal groups, respectively

As *Chryseobacterium* and *Pseudomonas* were the unique dominant bacterial genera in the roots of RW. A significant negative correlation was observed between *Chryseobacterium* and Glutathione, oxidized, while *Pseudomonas* showed a significant positive correlation with Galactinol and (–)–Deoxypodophyllotoxin. Additionally, *unclassified_f__Rhizobiaceae*, *Mycobacterium* and *Afipia* were the unique dominant bacterial genera in the roots of SW. Among them, *unclassified_f__Rhizobiaceae* exhibited a highly significant positive correlation with Cucurbitacin I and Cucurbitacin B, and a significant negative correlation with O–Acetylserine, Cis–Zeatin O–glucoside, O–Beta–D–Glucosylzeatin, 5–Hydroxyconiferyl alcohol, Xanthosine, GDP–glucose, DADP, and Cinnamaldehyde; Meanwhile, *Mycobacterium* showed a highly significant positive correlation with Cucurbitacin I and Cucurbitacin B, and a significant negative correlation with O–Acetylserine, Cis–Zeatin O–glucoside, O–Beta–D–Glucosylzeatin, 5–Hydroxyconiferyl alcohol, Xanthosine, Abscisic Acid, Trehalose, GDP–glucose, Trans–Cinnamaldehyde, DADP, Cinnamaldehyde, and Aldosterone 18–glucuronide; Moreover, *Afipia* exhibited a significantly positive correlation with Cucurbitacin I and a significantly negative correlation with Cinnamaldehyde, 5–Hydroxyconiferyl alcohol, and GDP–glucose (Fig. 9A).

In terms of fungal genera, *Plectosphaerella* exhibited a significantly negative correlation with Cucurbitacin I; and *unclassified_o__Sordariales* showed a significantly negative correlation with Allantoic Acid; Also, *unclassified_f__Lasiosphaeriaceae* displayed a significantly positive correlation with O–Beta–D–Glucosylzeatin; Meanwhile, *unclassified_k__Fungi* presented a significantly positive correlation with 5–Hydroxyconiferyl alcohol; *unclassified_o__Branch06* showed a significant positive correlation with Stachyose.

As *Neocosmospora* and *unclassified_f__Lasiosphaeriaceae* were the unique dominant endophytic fungal genera in the roots of RW. Among them, *Neocosmospora* exhibited a significantly positive correlation with FMNH2 and O–Acetylserine; And *unclassified_f__Lasiosphaeriaceae* showed a significantly positive correlation with O–Beta–D–Glucosylzeatin. In contrast, *unclassified_p__Ascomycota*, *unclassified_o__Sordariales*, *Talaromyces*, *Podospora*, and *Serendipita* were the unique dominant endophytic fungal genera in the roots of SW. Among them, *unclassified_p__Ascomycota* displayed a significantly positive correlation with 3’,5’–Cyclic GMP, Cucurbitacin I, and Cucurbitacin B; Also, it showed a significantly negative correlation with Lactose, Trehalose, GDP–glucose, 5–Hydroxyconiferyl alcohol, Xanthosine, Cis–Zeatin O–glucoside, O–Beta–D–Glucosylzeatin, Aldosterone 18–glucuronide, Trans–Cinnamaldehyde, DADP, Cinnamaldehyde, and Heme; Meanwhile, *unclassified_o__Sordariales* exhibited a significantly negative correlation with Allantoic Acid; And *Talaromyces* showed a significantly negative correlation with Xanthosine and O–Acetylserine; *Podospora* also displayed a significantly negative correlation with FMNH2, S–Adenosylhomocysteine, Maltotriose, Raffinose, (–)–Deoxypodophyllotoxin, Stachyose, Trans–Cinnamaldehyde, and Abscisic Acid; Moreover, *Serendipita* exhibited a significantly negative correlation with Cucurbitacin B, Raffinose, (–)–Deoxypodophyllotoxin, Stachyose, and Heme (Fig. 9B).

## Discussion

Endophytic microbial communities are the plant’s second genome, playing an important role in promoting plant growth, development, and defense [45]. Endophytic microorganisms can enhance plant stress resistance by directly inducing the immune system and influencing plant growth through the secretion of plant hormones, such as cytokinins, gibberellins, and auxins, as well as the production of secondary metabolites [46, 47]. Among them, endophytic bacteria contribute positive correction with plant nutrition by promoting nutrient absorption and the release of metabolites or enzymes in the roots. Additionally, specific metabolites could be produced by endophytic fungi which had a positive impact on plant growth and the potential functions against plant pathogens [48, 49]. Previous studies have found that plant disease resistance is an important factor driving changes in endophytic microbial communities [16], with significant differences in endophytic microorganisms between resistant and susceptible plants [16–18]. The main difference between resistant and susceptible plants also lies in whether the host can recognize pathogen invasion and the speed of the response to the invasion [50–52]. In susceptible plants to diseases, slow response and weak defense signals may lead to pathogen spreading throughout the plant and damages can be caused. On the contrary, not only rapid response, but also strong defense signals can be detected in resistant plants to diseases. Based on this background, we conducted an in-depth analysis of the endophytic microbiome and metabolome in *Fusarium* wilt-resistant and susceptible watermelon varieties.

In this study, distinct microbial signatures were identified between *Fusarium* wilt-resistant (RW) and susceptible watermelon (SW) varieties. The root endophytic bacterial communities of RW were uniquely dominated by *Chryseobacterium*, *Pseudomonas*, *Delftia*, *Lechevalieria*, *unclassified_f__Methylophilaceae*, and *Tahibacter*, while fungal communities were characterized by *unclassified_p__Basidiomycota*, *Neocosmospora*, *unclassified_f__Lasiosphaeriaceae*, and *Edenia*. In contrast, SW roots exhibited specific enrichment of *unclassified_f__Rhizobiaceae*, *norank_f__norank_o__Microtrichales*, *Mycobacterium*, and *Afipia* in bacterial populations, along with *unclassified_p__Ascomycota*, *unclassified_o__Sordariales*, *Talaromyces*,* Podospora*, and *Serendipita* in fungal communities. Previous studies had confirmed that *Pseudomonas* was a typical biocontrol bacterial genus for promoting plant growth by enhancing plant nutrient absorption and secreting biological compounds against plant pathogens. For example, it had been reported in maize [53], tomatoes [54], and black pepper [55]. Meanwhile, *Pseudomonas* was also reported that it was effective in preventing watermelon wilt disease [56]. Additionally, *Chryseobacterium* was also reported that it was frequently appeared in plant roots with a potential function in antagonizing plant pathogens [57]. Comparative analysis revealed that RW not only harbored higher abundances of these antagonistic bacteria but also maintained a more stable endophytic microbial network architecture. This stability was evidenced by significantly stronger positive interspecies connectivity within RW’s microbial community, suggesting enhanced cooperative interactions that may contribute to pathogen suppression. Such network robustness contrasts with the fragmented microbial associations observed in SW, potentially explaining its disease susceptibility.

Additionally, significant differences of metabolites and functional characteristics also could be found in the roots between RW and SW. Particularly, in comparison with SW, more abundant upregulated metabolic pathways, such as Galactose metabolism, Phenylpropanoid biosynthesis, Phenylalanine, tyrosine and tryptophan biosynthesis could be detected in the roots of RW. Among them, Galactose metabolism plays a key role in supporting microorganisms that utilize galactose as a carbon and energy source; in turn, viable endophytic microorganisms in the roots can modulate the plant’s immune response. In addition, several sugars produced via Galactose metabolism—including sucrose, stachyose, and galactinol—have been shown to participate in plant defense by activating immune signaling pathways and enhancing structural barriers [58, 59]. Specifically, sucrose is recognized as a key signaling molecule in plant immune responses, triggering defense mechanisms against a variety of pathogens [60]. Stachyose has been reported to strengthen the cell wall of wheat, improving resistance to *Fusarium graminearum* invasion [61]. Galactinol, as a biosynthetic precursor for cell wall polysaccharides, contributes to reinforcing cell wall structure and regulating its function, thereby enhancing the plant’s overall defense capacity [62]. In comparison with SW, significant upregulation of sucrose, stachyose and galactinol all could be detected in the roots of RW.

Also, the Phenylpropanoid biosynthesis pathway, not only is a crucial pathway in plants biomass metabolism [63], but also is a vital component for promoting plants defense system. For instance, lignin, a secondary metabolite of the phenylpropane metabolic pathway, can enhance cell wall hardness, preventing mechanical damage and inhibiting pathogen e invasion [64]. As the degree of lignin thickening had been reported that it exactly related to wilt resistant abilities of watermelons [65]. We also found that the metabolites, such as Coniferyl Aldehyde, Coniferin, 5–Hydroxyconiferyl alcohol, 4–Coumaryl alcohol, which were in associated with vascular bundle cells for promoting host resist ability to pathogenic bacteria, were significantly upregulated in the roots of RW than those of SW. It suggested that lignification also was the important mechanism for RW with higher resistant ability to watermelon *Fusarium* wilt. The metabolic pathways of phenylalanine, tyrosine, and tryptophan biosynthesis are closely related to the plant cell wall and are also significantly associated with plant stress resistance. The expression levels of this metabolic pathways in RW roots were also significantly higher than those in SW roots. Simultaneously, significantly upregulated substance 3–Hydroxybenzoic Acid, also known as salicylic acid [SA], was also enriched in this pathway. Salicylic acid (SA), serving as a mediator between plants and microbes, is a crucial signaling molecule inducing plant disease resistance [66]. For example, exogenous SA treatment could selectively increase the abundance of beneficial microorganisms [67]. And the synthesis of SA in watermelon plants could enhance their resistance to *Fusarium* wilt [68].

All above results suggested that the endophytic microorganisms and metabolites in the roots of watermelon varieties exactly were closely associated with resistant ability to *Fusarium* wilt. The enrichments of antagonistic endophytic microorganisms and higher abundances of metabolites for promoting cell wall hardness in the roots of RW are the important mechanisms for its higher resistant ability to *Fusarium* wilt than those of wilt susceptible watermelon varieties.

## Conclusions

Endophytic microorganisms and metabolites in the roots of watermelon varieties exactly are closely associated with resistant ability to *Fusarium* wilt. The endophytic bacterial genera, such as *Chryseobacterium*, *Pseudomonas*, *Delftia*, *Lechevalieria*, *unclassified_f__Methylophilaceae*, *Tahibacter*, and the endophytic fungal genera, *unclassified_p__Basidiomycota*, *Neocosmospora*, *unclassified_f__Lasiosphaeriaceae*, *Edenia*, were the unique dominant bacterial and fungal genera in the roots of wilt resistant watermelon varieties. The results suggested that the endophytic microbial communities in the roots of wilt resistant watermelon varieties could be speculated as part of the antagonistic microbiome against watermelon wilt; Additionally, the significantly upregulated Galactose metabolism, Phenylpropanoid biosynthesis, Phenylalanine, tyrosine and tryptophan biosynthesis in wilt resistant watermelon varieties indicated that these pathways could contribute to resistance to Fusarium wilt. As a result, Galactinol, Sucrose, Stachyose, Coniferyl Aldehyde, Coniferin, 5–Hydroxyconiferyl alcohol, 4–Coumaryl alcohol and 3–Hydroxybenzoic Acid could be considered as the antagonistic metabolites in biocontrolling watermelon wilt. This study systematically reveals, for the first time, the synergistic defense mechanisms between root endophytic microbiome and metabolome during *Fusarium* wilt resistance formation in watermelon. Significantly, we have identified potential functional microorganisms, key metabolites, and critical pathways that actively contribute to these defense mechanisms. However, the specific functions of these potential antagonistic microorganisms and metabolites still need further validation. These findings provide a novel perspective for crop disease resistance research, transcending the limitations of traditional single-factor analytical paradigms, while establishing a methodological foundation for developing multi-omics integrated approaches in crop disease resistance regulation strategies.

## Supplementary Information

Supplementary Material 1.

## Data Availability

The raw sequencing data for endophytic bacteria and fungi have been deposited in the NCBI Sequence Read Archive (SRA). The bacterial sequence data (BioProject: PRJNA1080270) are accessible under the following SRA run accessions: SRR28088066, SRR28088073, SRR28088074, SRR28088075, SRR28088076, SRR28088077, SRR28088078, SRR28088079, SRR28088081, SRR28088082, SRR28088083, SRR28088084, SRR28088085, SRR28088086, SRR28088087, SRR28088088, SRR28088089, SRR28088090. The fungal sequence data (BioProject: PRJNA1080342) are accessible under SRA run accessions: SRR28089671, SRR28089689, SRR28089690, SRR28089691, SRR28089692, SRR28089693, SRR28089694, SRR28089695, SRR280896978, SRR280896979, SRR280896980, SRR280896981, SRR280896982, SRR280896983, SRR280896984, SRR280896986, SRR280896987, SRR280896988.
